# Effectiveness of Photodynamic Therapy as Antiseptic Measure for Oral Cavity and Pharynx: A Systematic Review

**DOI:** 10.3390/dj11080192

**Published:** 2023-08-10

**Authors:** Diana Sologova, Marina Petukhova, Polina Podoplelova, Dinislam Davletshin, Anna Firsova, Andrey Grishin, Mikhail Grin, Nikita Suvorov, Yuriy Vasil’ev, Sergey Dydykin, Elena Rysanova, Victoria Shchelkova, Svetlana Tarasenko, Ekaterina Diachkova

**Affiliations:** 1 Department of Oral Surgery of the Institute of Dentistry, I.M. Sechenov First Moscow State Medical University (Sechenov University), 119048 Moscow, Russia; marina.petukhova2014@ya.ru (M.P.); pdpelovapolina@gmail.com (P.P.); davletshin.dinislam@gmail.com (D.D.); ann.f.710@ya.ru (A.F.); prof_tarasenko@rambler.ru (S.T.); secu2003@mail.ru (E.D.); 2 Maxillofacial Surgery Department, I.M. Sechenov First Moscow State Medical University (Sechenov University), Trubetskaya Street 8\2, 119991 Moscow, Russia; dr.grishin@mail.ru; 3 Department of Chemistry and Technology of Biologically Active Compounds, Medicinal and Organic Chemistry, Institute of Fine Chemical Technologies, MIREA-Russian Technological University, 86 Vernadsky Avenue, 119571 Moscow, Russia; michael_grin@mail.ru (M.G.); suvorov.nv@gmail.com (N.S.); 4 Department of Operative Surgery and Topographic Anatomy, I.M. Sechenov First Moscow State Medical University (Sechenov University), Trubetskaya Street bldg. 8\2, 119435 Moscow, Russia; y_vasiliev@list.ru (Y.V.); dydykin_s_s@staff.sechenov.ru (S.D.); 5 Moscow Regional Research and Clinical Institute, Street Schepkina 61/2, 129110 Moscow, Russia; e.rysanova@monikiweb.ru (E.R.); victoria.shchelkova@yandex.ru (V.S.)

**Keywords:** photodynamic therapy, antibacterial, bactericide, oral cavity, microflora, photosensitizers, randomized clinical trials

## Abstract

Background: The complex traditional treatment of inflammation diseases in oral cavity includes the prescription of antibiotic and antiseptic therapy. This systematic review aims to evaluate the effect of photodynamic therapy as a part of management of inflammatory diseases in oral cavity; Methods: The study is presented in accordance with the preferred reporting points for systematic reviews and meta-analyses (PRISMA). This systematic review was conducted using electronic databases such as Medline PubMed, Scopus and the Cochrane Central Register of Controlled Trials. All the studies in this systematic review, were randomized, the risk of bias 2 (ROB 2) were assessed; Results: Considering the inclusion and exclusion criteria, we included 10 randomized clinical trials, published up to 2023 investigating the application of photodynamic therapy as a part of management of inflammatory diseases in oral cavity. The diode laser was used in the oral cavity in the zone of inflammatory process (gingivitis, mucositis, periimplantitis, marginal periodontitis, abscess, periostitis, osteomyelitis etc.) in nine studies or in the zone before surgical procedures in one study; Conclusion: Based on the results of clinical studies, it can be stated that photodynamic therapy shows good results for operations performed in the oral cavity and pharynx.

## 1. Introduction

Periodontal diseases are an important aspect of study in dentistry. It is known that periodontal microflora becomes a frequent cause of their occurrence. These bacteria are aggressive towards tooth-supporting tissues [1]. Traditional non-surgical treatment has a number of drawbacks, such as: tissue damage, insufficient antiseptic treatment, the problem of treating hard-to-reach areas [1]. The high adhesive and invasive ability of bacteria, toxic effects, protective properties—all this causes difficulties in the treatment process, resulting in chronic periodontitis [2]. Thus, complex treatment is impossible without elimination of periodontal microflora and neutralization with the help of anti-inflammatory and antimicrobial drugs [2]. It is also important to note that, despite the positive results at the beginning of treatment, in the treatment of this pathology, relapses often occur, so this aspect is one of the important negative components of the complex of therapeutic and prophylactic manipulations in the treatment of this pathology [1].

In this regard, of particular interest is the method of photodynamic therapy, which has been widely used in recent decades for the adjunctive treatment of various diseases of inflammatory genesis, including in dentistry.

Photodynamic therapy consists of photosensitizer, light and oxygen [3]. The basis of photodynamic therapy is the ability of some chemical preparations, called photosensitizers, to sensitize biological tissues in the presence of oxygen to the effects of light radiation of a certain wavelength [4]. As a sensitizer in cells, both natural metabolites—chlorophyll, flavins, porphyrins, bilirubin (endogenous sensitizers), and a wide range of exogenous substances entering the cells—visible light acceptors (dyes, aromatic hydrocarbons) can act [5]. There is an excitation of the sensitizer, a photochemical reaction occurs, the result of which is the release of singlet oxygen or free radicals [6,7]. A particular case of photosensitized processes is the photodamage of biological systems in the presence of sensitizers with the participation of molecular oxygen—the so-called photodynamic action [6,7]. The substance (photosensitizer) is applied inside the periodontal pocket and is activated by a light source. A photosensitizer bonded to bacteria can be activated by light of the appropriate wavelength in the presence of oxygen to generate singlet oxygen and free radicals. Free radicals are cytotoxic to microorganisms, result in damage to the cytoplasmic membrane and DNA. The name of this process is antimicrobial photodynamic therapy [8,9].

Laser radiation is widely used as a light source for the application of photodynamic therapy, which not only activates photosensitizers, but can also be used as an additional mechanical treatment of tissues [10]. Lasers have some unique characteristics, such as being monochromatic, coherent, controllable wavelength and high power for activating photosensitizers [10]. The most common lasers are diode lasers (wavelength 650–1000 nm) due to their economic convenience and portability as compared to erbium lasers (Er: YAG), neodymium-doped yttrium aluminum garnet (Nd: YAG) and chromium-doped yttrium scandium gallium garnet (Cr: YSGG) [11,12,13].

In the articles we’ve reviewed, special attention is paid to the mechanism of photodynamic therapy. As for the effectiveness, the result of this method does not depend on the spectrum of sensitivity of microorganisms to antibiotics, and photochemical exposure is able to destroy the microbial matrix and penetrate into the subsurface layers of the epithelium [5,14]. It is also worth paying attention to the fact that this method of treatment using an LED, laser and photosensitizers is used not only for the treatment of periodontal diseases, but can also be actively used for antibacterial treatment, for example, in the oral cavity and oropharynx, for the treatment of inflammatory diseases [14,15,16]. The advantage of the technique of laser photodynamic therapy is that the death of the microflora is achieved in a short period of time and at the same time any damage to the surround tissues is excluded. It is important to notice that it is this safety factor that allows the use of photodynamic therapy in hard-to-reach areas, for example, in dentistry with endodontic treatment in the root canals of teeth [17,18,19,20,21]. It is also noted that this method can be an alternative to the use of modern antibiotics and antiseptics, due to the properties of hypersensitivity of pathogenic bacteria. Studies of this treatment method have not been completed, it is important to understand the body’s reaction to the laser action, the safety of the procedure, the resistance of periodontal microflora and the development of bacterial resistance to reactive oxygen species [18,21].

This systematic review aims to evaluate the effect of photodynamic therapy as a part of management of inflammatory diseases in oral cavity. This topic has been repeatedly covered in various studies [15,17,22,23,24,25,26,27,28,29,30,31,32,33]. Photodynamic therapy is considered as type of adjunctive treatment in various inflammatory diseases in oral cavity, however, there are many unexplored aspects that are disclosed in this review. We analyzed all existed systematic reviews on this subject and compose a new systematic review dedicated to application of photodynamic therapy as antiseptic and antimicrobial measure in oral cavity and pharynx at all. In this review, selection and exclusion criteria cover various aspects, such as analgesics, potential and existing pregnancy of patients, general oral health, and others, which allows narrowing the study area and providing more accurate results.

In this review, more attention was paid to the selection and exclusion criteria. Compared to similar articles, this review covers various aspects. If we consider the criteria, the important point was the age of the patients, pregnancy or lactation. Special attention was paid to concomitant diseases, concomitant medications and the condition of the dental system. The review also draws attention to the conduct of research, namely the evaluation of the results. All this allow to narrow down the range of study and give more accurate results.

## 2. Materials and Methods

This study was prepared referring to the Cochrane tool to assess the risk of bias in randomized trial, and performed according to the Preferred Reporting Items for Systematic Reviews and Meta-Analyses (PRISMA) [34].

This systematic review is registered in the International prospective register of systematic reviews (PROSPERO).

The registration number: CRD42022348087.

### 2.1. Eligibility Criteria

In this systematic review, the literature search was based on the PICO (patient, intervention, comparison, and outcome) format.

Types of participants: patients not younger than 18 and not older than 80 years.

Types of interventions: the use of photodynamic therapy as a part of management of inflammatory diseases in oral cavity and pharynx.

Types of control: as for comparisons, we included treatment without using aPDT.

Outcome: assessment parameters depend on type of inflammation disease in oral cavity.

Types of studies: RCT’s.

Only trials published in English were selected. Conference abstracts, reviews, commentaries and case reports were excluded.

### 2.2. Sources of Information

A literature search was conducted on 4 electronic databases up to 1 May 2023, Medline PubMed, Scopus, and the Cochrane.

### 2.3. Search Strategy

The search terms describe the PECO components:

P (participants)—patients with inflammatory diseases in oral cavity or pharynx;

E (exposure)—patients with inflammatory diseases in oral cavity or pharynx who were treated with adjunctive use of aPDT;

C (comparison)—patients with inflammatory diseases in oral cavity or pharynx who were treated without aPDT;

O (outcome)—to evaluate the effectiveness of PDT in patients with inflammatory diseases in oral cavity or pharynx based on assessment of follow criteria: microbial load, pocket depth, clinical attachment level (CAL), bleeding on probing (BOP), plaque index (PI), gingival index (GI).

Exclusion and Inclusion Criteria:

Inclusion criteria:

- Randomized clinical trials;
- Participants are older 18 years and younger 80 years with the indication for oral surgery with/or inflammatory diseases of oral cavity and pharynx;
- Use of photodynamic therapy as antiseptic method before operations in oral cavity or the part of management of inflammatory diseases of oral cavity and pharynx.

Exclusion criteria:

- Other types of research;
- Participants are younger than 18 years or older than 80 years old;
- Antibiotics taking for last month, anti-inflammatory drugs or pain-killers taking for last 5 days;
- Severe comorbidity proof;
- Pregnancy or lactation;
- Fungal infection;
- Acute viral or bacterial infections of other localization;
- Other conditions that are contraindications for laser use.

The filters were used: full texts, humans, clinical trials, randomized clinical trials, 11 years publication date between 2013 and 2023.

The search algorithms were used for the search in the Medline PubMed.

The following search of terms were used for all four data bases (Medline PubMed, Scopus, and the Cochrane): «photodynamic therapy», «antimicrobial photodynamic therapy» «antibacterial», «bactericide», «oral cavity», «microflora», «photosensitizers», «randomized clinical trials».

Medline/PubMed/Scopus/the Cochrane.

(«Photodynamic therapy» OR «antimicrobial photodynamic therapy») AND («antibacterial» OR «bactericide» OR «antimicrobial») AND «oral cavity» AND «microflora» AND «photosensitizers» AND «randomized clinical trials».

The date range of included articles was 2013 to 2023. We reviewed published systematic reviews and meta-analyses to ensure that the topic is relevant and that there is no duplication or plagiarism.

Two groups contained three researchers (A.F., P.P., M.P; D.D., D.S., N.S) independently searched databases for the above criteria. In case of some disagreement for all group two independent researchers helped to have consensus (D.E.; Y.V.).

### 2.4. Selection of Studies

The date range of included articles was 2013 to 2023. Three researchers independently searched databases for the above criteria.

### 2.5. Data Collection Process and Items

The following data were recorded for each selected study: the source of publication (author, year, and journal of publication), the design of the study, participants (sample size, age, sex, and comorbidities) and outcomes (changing of assessed criteria as the base for effectiveness evaluation for PDT in patients with the inflammatory diseases of oral cavity and pharynx).

Three independent authors two times performed the data extraction and collection process. Some disagreements between authors were resolved.

### 2.6. Study Risk of Bias Assessment

To exclude Bias Assessment were conducted a study with Cochrane “Risk of Bias tool” (RoB 2.0) (Higgins 2019) [35] method:

- The randomization process;
- Deviations from intended interventions;
- Missing outcome data;
- Measurement of the outcome; and
- Selection of the reported result.

The bias were analyzed independently by two authors. Also were applied the tool for each study and justifications for judgements of risk of bias (low; high; some concerns). Authors resolved any disagreements in the assessment of risk of bias between each other. Each item was assessed (low; high; some concerns) by authors in the risk of bias table.

## 3. Results

### 3.1. Study Selection

**Figure 1:**
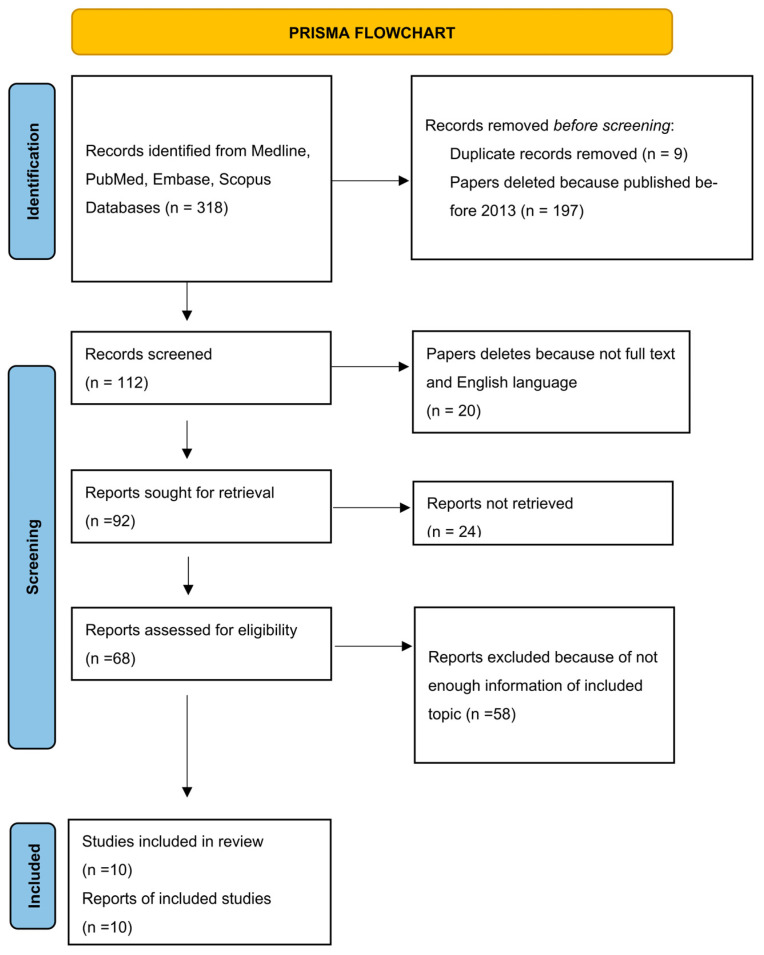
Flow diagram describing the selection process according to the Preferred Reporting Items for Systematic Reviews and Meta-Analyses (PRISMA) recommendations [34].

The study selection flow chart (Figure 1) [34] included 318 results from electronic databases. After the exclusion of articles published earlier than 2013 and duplication records removed, we obtained 112 studies. Then, we filtered 20 articles without full text and/or not written in English and received 92 results. We excluded 24 articles due to not retrieved reports. Fifty-eight reports were excluded because of lack of information on included topic. The final amount of publications is 10 (Table 1).

### 3.2. Study Characteristics

Each of the 10 studies was RCT and included studies of patients without comorbidities and not under anti-inflammatory pain killers within the last 5 days or/and not under antibiotics for the last month. The necessary moment was the absence of other methods of antiseptic in the test group of patients.

Diode lasers with a wavelength from 660 nm to 940 nm were used across studies. The diode laser was used in the oral cavity in the zone of inflammatory process (gingivitis, mucositis, periimplantitis, marginal periodontitis, abscess, periostitis etc.) in nine studies or in the zone before operations (tooth extraction, dental implantation, bone plasty etc.) in one study. Modes and duration were different. In studies, the authors compared a pockets depth and the number of pathogenic bacteria before and after the surgery.

The periodontal probing depth (PPD) of periodontal pockets was decreased after the aPDT in eight included studies [2,4,36,38,39,40,41,43]. The sulcus fluid flow rate (SFFR) showed significantly lower mean values in the aPDT group in one study [40]. The clinical attachment level (CAL) was significantly decreased in aPDT group in four articles [2,36,39,41]. The level of Streptococcus oralis after aPDT was significantly decreased in one article [42]. The level of 40 subgingival species was decreased in one study after the aPDT [38]. The reduction of halitosis in patients with chronic periodontitis was noted in one article [41].

### 3.3. Risk of Bias within Studies

All 10 studies included in systematic review were randomized, risk of bias 2 (Rub 2) was used (Table 2).

## 4. Discussion

In recent years, photodynamic therapy (PDT) has attracted an increasing attention due to its ability to suppress the activity of drug-resistant bacteria.

Bacterial infections in oral cavity are very common in clinical practice. Systemic and local antimicrobials are the main methods of treating these diseases. However, in conditions of rapid growth and drug resistance, the PDT method is universal [6].

Antimicrobial photodynamic therapy is a modern treatment method aimed at deactivating microorganisms responsible for the occurrence and progression of oral diseases [8]. PDT is defined as an oxygen-based photochemical reaction involving a photosensitizer, a light source, and oxidizing molecules [10]. Due to its high antibacterial potential, this method can be used in the treatment of periodontitis, peri-implantitis, as well as in endodontic therapy. Unlike antibacterial therapy, the PDT method does not produce resistance to microorganisms, so this procedure can be repeated many times without any consequences for the patient’s body and general health [2].

Photodynamic therapy is a method based on the activation of exogenous photosensitizing agents using a light source [4].

If we consider photosensitizers, then several generations can be distinguished. The very first drugs appeared in the 1960s—derivatives of hematoporphyrin. These include the gold standard PDT—Photofrin^®^. However, despite its effectiveness, there are certain limitations in its use—the accumulation in the body and the absorption layer of light, which is able to be scattered and absorbed by the cells of the body [45,46].

To overcome these shortcomings, second-generation PS were created. The wavelength range for the activation of these synthetic drugs varies from 600 to 800 nm, thereby allowing them to penetrate deeper into the tissues. However, the presence of an optimized spectrum does not ensure selectivity. Also, second-generation drugs are rapidly eliminated from the body, thereby reducing the time of photosensitization and the effectiveness of treatment [45,46].

Scientists achieve the greatest selectivity in relation to tissues in third-generation drugs, trying to build a receptor-target relationship. An equally important point is the expansion of light absorption—the desire for infrared absorption. Also among the new PS there are inorganic substances that have the necessary potential in biotechnology and nanotechnology [45].

Due to the lack of protocols and conflicting results of the studies, the evidence for a PDT is yet insufficient for clinical recommendations [47]. However, scientists and medical practitioners investigate new therapeutic approaches for sustained treatment outcomes. As additional treatment of periodontitis, the complexed use of diode laser and indocyanine green can be used [36].

PDT involves the introduction of a photoactive dye that is capable of producing reactive oxygen species (ROS) when irradiated with light. So, when a dye absorbs a photon, an electron is transferred from its ground state to an electronically excited state, which returns energy along three main paths. When light energy is absorbed at the corresponding wavelength, the photosensitizer transitioned from a low-energy singlet ground state to a triplet state with higher energy [48].

Antimicrobial photodynamic therapy can solve the problems of conventional antimicrobial therapy and can be used as an additional to the main mechanical treatment [49]. The photosensitizer and the optical fiber placed in the periodontal and peri-implant pockets could reach the root or implant surface before activation with the laser light [50]. The big amount of randomized controlled clinical trials has shown that a-PDT and scale root planning (SRP) can cause significant enhancements in PPD and clinical attachment level than SRP alone [32,33,51,52,53]. The aPDT properties against bacteria and virus agents could be realized with different lasers and photosensitive substances. In aPDT, the photosensitizers employed are toluidine blue O, methylene blue, erythrosine, chlorine E-6, hematoporphyrin [54].

The method of implant surface scaling (ISS) plus photodynamic therapy was assessed in some trials in the treatment of peri-implantitis and mucositis [15,31,55,56,57]. Based on the above studies, aPDT can be used as an alternative to antibiotics and as adjunctive treatment of peri-implantitis. The combination of traditional mechanical treatment of peri-implantitis and aPDT reduces bacterial load and may be an alternative to antibiotics [57,58].

In comparison to other therapies, a-PDT is less invasive and can be used for all patients, except for those with hypersensitivity to light. It can be used more frequently than antimicrobials because a-PDT does not cause bacterial resistance [15]. Moreover a-PDT can inactivate endotoxins that are produced by Gram-negative bacteria [59,60] and can affect wound healing [61].

### 4.1. Review Study Discussion

The study of the effectiveness of antimicrobial photodynamic therapy (aPDT) was conducted by the authors of an article published in 2014. Basically, the mechanism of action of reactive oxygen species (ROS) was studied. In their work, the authors used a combination of toluidine blue O (TBO) and red LED (LED) to conduct 4 experiments. The results were as follows: the best antibacterial effect and the largest amount of reactive oxygen species were observed when using the combination of TBO and LED, while the cytotoxic effect with the additional use of LED did not have a particularly significant difference. The cytotoxicity results of aPDT were close to the cytotoxicity results of antiseptics used in dentistry. An important point of the article was that the authors drew attention to the safety of photodynamic laser therapy. This method of treatment significantly suppressed the formation of plaque, while without adversely affecting surrounding tissues and teeth [42].

Since the article examined the effects of aPDT only on *S. oralis*, the authors of a randomized clinical trial in 2017 studied the antibacterial effect when exposed to other bacteria involved in the formation of plaque (Treponema denticola, Actinomyces gerencseriae and Actinomyces oris). The authors studied the effect of antimicrobial photodynamic therapy on the microbiological state of tissues. As a result of surgical treatment of Severe Chronic Periodontitis using aPDT in one episode with a diode laser and a phenothiazine photosensitizer, the study showed a reduction in inflammation. It is important to note that the Flap treatment has led to a decrease in the number of pathogenic periodontal microorganisms and an increase in the number of species to the favorable microflora of the oral cavity. In addition, the article stated that this treatment method can be used repeatedly due to the low risk of bacterial resistance to reactive oxygen species [38]. Thus, in order to supplement the information about the resistance of microflora, the authors of the article, published in 2022, conducted a study on this topic.

Colleagues noted that PDT has a high antibacterial potential. As a result, it can be used as adjunctive treatment of various diseases of the oral cavity. In addition, studies have shown that, unlike antibiotics, bacteria do not develop resistance to repeated photodynamic therapy methods. There is also a lot of evidence that PDT can destroy biofilm, further reducing the viability of bacteria and increasing sensitivity to antibiotics [2].

The authors of the article published in 2014 were also interested in this method of additional processing. The authors, comparing the results of treatment of chronic periodontitis with the additional use of aPDT, noted that after 3 and 6 months there was a statistically significant decrease in parameters in the group after antibacterial photodynamic therapy (aPDT) in addition to scaling and root planing (SRP). The assessment was carried out according to the indicators of pocket depth and the clinical attachment levels, gingival index and gingival bleeding index, halitosis. The authors concluded that aPDT is a useful supplement to SRP in the non-surgical treatment of chronic periodontitis [41].

In an article published in 2022, non-surgical periodontal treatment of chronic periodontitis using erbium laser and a-PDT as an adjunct to SRP was considered. According to the results of the study, the authors claim that the result of using an antibacterial photodynamic system is good. However, the additional use of the laser showed a better result [39]. Thus, the use of photodynamic therapy in conjunction with a diode laser is actively used in various types of dental manipulations. In addition to treating diseases of the oral mucosa, dentists studied the effect of a diode laser in the root canals of teeth.

In a study of colleagues conducted in 2021, a diode laser was used after the usual irrigation of necrotic teeth in the first and second admission. Intra-channel laser irradiation was carried out in a pulsed mode with circular movements in order to reduce the heating of the dentin, thereby not damaging the surrounding periodontal tissues. The temperature of the channel wall immediately decreased when a laser connected to an activated fiber optical tip with a diameter of 200 microns was rapidly fed from the apical to the coronal direction. Consequently, the tissues surrounding the tooth are only slightly affected, and the peri radicular tissues are not damaged. The authors concluded that the use of a diode laser after conventional irrigation can reduce postoperative pain in single-root necrotic teeth with a PAI rating of 3 or 4 after root canal treatment carried out in two medical visits. The research results indicate that diode lasers can be used to treat root canals, providing patient comfort [44].

Despite the large number of articles with positive results when using antimicrobial photodynamic therapy, there are also works with neutral results. In an article published in 2019, the authors used antibacterial photodynamic therapy (aPDT) and a diode laser in the non-surgical treatment of chronic periodontitis. Comparing 2 processing methods (scaling and root planning (SRP) and SRP + aPDT in combination with a diode laser), the authors concluded that there were no significant differences between the groups after 3 months. Relative attachment level, probing depths and gingival recession had similar results in the two groups. The only different parameter was the sulcus fluid flow rate. The result showed a lower value after 2 weeks in the group where additional aPDT treatment was used. The authors are inclined to believe that these results could have been obtained as a result of insufficient power, concentration, repetitions or irradiated time. Under the conditions carried out during the study, the indicators for effective reduction of the number of bacteria may not have been achieved [40].

In an article published in 2019, the authors described and compared the long-term clinical effects of a diode laser and photodynamic therapy as additional treatments for chronic periodontitis in combination with scaling and root planning. The results of the present study did not reveal any clinical benefit after the use of diode or photodynamic laser therapy in combination with conventional SRP compared with mechanical therapy solely in terms of probing depth, CAL and bleeding during probing 6 months after treatment. There was only a tendency to a greater decrease in PD in the group of diodes for deep pockets after 3 months [4].

The absence of a significant difference in the results of the study, which was conducted in 2018, also casts doubt on the effectiveness of aPDT treatment. In this article, the authors provided the results after a single application of the antibacterial photodynamic system as an adjunct to open flap debridement at the time of treatment of peri-implantitis. The results were monitored for 12 months after the operation. Peri-implant plaque index, bleeding on probing, pocket depth and marginal bone level were evaluated. The average value of all indicators in all groups was significant if when considering the indicators from baseline to the result after 6 months. The group in which additional photodynamic therapy was used initially had a better indicator. However, after 12 months, the results in both groups were practically the same. The results equalized by the end of the study and did not have a significant difference. The authors suggest that such a result of the study could be due to a number of factors that were not taken into account in the exclusion criteria. According to the data on the participants, about 50% of patients smoked at the time of the study, and 36% of patients were prediabetic. As stated in this article, both the first and second factors stimulate the secretion of proinflammatory cytokines that cause osteolysis of the alveolar bone. It is also important to consider that the hyperglycemia can reduce the activity of the cell mediated immunity [43].

Among the new scientific papers written in 2023, it is important to single out a randomized clinical trial, during which the authors increased the sample of microbiological analysis, and also studied the change in the depth of gingival pockets. According to the results of the study, it was concluded that the combination of full-mouth ultrasonic subgingival debridement (FMUD) together with a diode laser and indocyanine green (ICG-aPDT) could not show high results, except for selective clinical and microbiological indicators. The authors noted in the article the high rate of pocket closure when using a diode laser. As for the results of microbiological analysis, a significant decrease was observed for two types of bacteria (Aggregatibacter actinomycetemcomitans and Parvimonas micra), but the result did not persist for a long time. In conclusion, the authors drew attention to the fact that randomized clinical trials with the possibility of changing laser parameters are still needed to confirm these results [36].

In addition, the use of photodynamics in combination with the main treatment method shows the best treatment results [4,36,37,38,39,40,41].

### 4.2. Limitations

We did not focus on any specific inflammatory disease of the oral cavity or pharynx, while the effectiveness of photodynamic therapy in various nosology can vary greatly. In addition, we analyzed all eligible studies without examining the effectiveness of a single laser type or regimen. Thus, despite the rather strong limitations for inclusion of studies in the analysis, the results are general and can serve as a guide for subsequent targeted systematic reviews and meta-analyses.

## 5. Conclusions

Thus, the antimicrobial photodynamic therapy can be used as adjunctive method in operations performed in the oral cavity and oropharynx for the treatment of inflammation diseases, stimulating the regeneration of tissues and microflora. Antimicrobial photodynamic therapy reduces bacterial load related to inflammation diseases and may be considered as an alternative to antibiotics. aPDT is a beneficial and promising therapeutic method, however, further high-quality RCTs focused on the standardized aPDT parameters are needed.

## Figures and Tables

**Table 1:** Characteristics of included studies—randomized controlled trials.

	Author (Name, Last Name), Year	N of Patients (Total, in Each Group)	Patients Age (Mean, Median)	Assessment Criterion	The Intervention Group (Test Group)	The Control Group	Laser Treatment (Mode), Photosensitizer	Location Use/Disease	Results
1	Marco Annunziata, 2023 [36]	24: 12 + 12	18–80, 25	- Probing death (PD) - Recession - (REC) - Clinical attachment level (CAL) - Bleeding on probing (BOP) - -Plaque index (PI) - Patient-reported outcome measures (PRPMs)	ICG-aPDT with an 810 nm diode laser (ICG-indocyanine green photosensitizer)	Irrigating the pockets with the photosensitizer solution and carrying inside the optical fibre with the laser kept it turned off mode	- 300-μm bulb optical fibre of the 810 nm diode laser unit set at 300 mw in pulsed mode - ICG-indocyanine green photosensitizer	Periodontitis	In the Test group: at 6 months for a higher PD reduction in initial deep pockets (PD ≥ 6 mm) and a higher percentage of closed pockets (PD ≤ 4 mm/no bleeding on probing). - Reduction in Aggregatibacter actinomycetemcomitans and Parvimonas micra levels in the test group at 3 months.
2	Kaveri Kranti Gandhi, 2019 [37]	30	30–60	- Gingival Index - Probing depth - Clinical attachment level - Porphyromonas gingivalis - Aggregatibacter actinomycetemcomitans	Test group1: (Scaling and root planning (SRP) + PDT) Test group2 (SRP + low level laser therapy (LLLT))	Scaling and root planning (SRP) alone	- -GaAIAs diode laser 100 MW 810 nm - ICG-indocyanine green photosensitizer	Chronic periodontitis	The test groups showed significantly higher reductions in Gingival Index, probing depth, and clinical attachment level
3	Sérgio H. L. Martins, 2017 [38]	40	≥35 years	- Probing pocket death - Levels of 40 subgingival species	Patients with severe chronic periodontitis (SCP) were treated with aPDT + ST (surgical periodontal treatment)	Patients with severe chronic periodontitis treated only with ST (surgical periodontal treatment)	Red Laser 70 mW of power, and a power density of 28 mW/cm^2^ - Photosensitizer: application of phenothiazine chloride solution	Severe chronic periodontitis	- Test Group presented a significantly higher decrease in PPD than Control Group at 90 days after surgical therapy. - Test Group demonstrated less periodontal pathogens of red complex (Treponema denticola)
4	Zoran Arsić 2022 [2]	25	30–70	- Plaque index (PI) - Bleeding on probing (BOP) - Probing depth (PD) - Clinical attachment level (CAL)	The test group was treated by NSPT (non-surgical periodontal treatment) combined with aPDT	The control group was treated by NSPT (non-surgical periodontal treatment) applied alone	- 660 nm diode laser powered by 100 mW Fibers 450 µm - The photosensitizer used was phenothiazine chloride	Periodontitis	NSPT combined with aPDT led to a statistically significant improvement of both clinical parameters and microbiological status compared to NSPT applied on its own
5	Subasree Soundarajan 2022 [39]	36	18–70	- Plaque index (PI) - Gingival index (GI) - Probing depth (PD) - Clinical attachment level (CAL)	Group III—SRP (scaling and root planning) followed by antimicrobial Photodynamic therapy using diode laser	Group I—Scaling and root planing (SRP) alone Group II—SRP followed by application of Er, Cr: YSGG laser	- 660 nm diode laser, 70 mW - The photosensitizer: methylene blue	chronic periodontitis	PI, GI PD, and CAL significantly improved at 3 months follow up compared to baseline in Group II and Group III with *p* < 0.05
6	Fotios Katsikanis, 2019 [4]	21	48.2 ± 8.2	periodontal pocket depth	Diode group-SRP with diode laser Photodynamic group- SRP with photodynamic therapy	The control group: SRP (scaling and root planning) alone	- low-level GaAlAs 670 nm diode laser (PDT), - Diode laser (940 nm) - The photosensitizer: methylene blue	severe periodontal disease pocket depth (PPD) of ≥5 mm	- There was no statistically significant difference regarding PD and BOP between groups. - There was only a tendency for greater reduction of PD in the diode group for deep pockets at 3 months, but not statistically significant
7	Greta Hill, 2019 [40]	20	61.1	- Relative attachment level (RAL) - probing depths (PD) - gingival recession (GR) - bleeding on probing (BOP) - sulcus fluid flow rate (SFFR)	ICG-based aPDT with a Diode laser	Patients were treated with scaling and root planning of the affected teeth	- diode laser at 808 nm 100 mW at 2 kHz - The photosensitizer: indocyanine green	chronic periodontitis	- none of the assessed parameters showed significant differences between the test and control groups - the SFFR showed significantly lower mean values in the aPDT group
8	Betsy Joseph, 2014 [41]	88	Control group 38.4 ± 9.6 years Test group 40.8 ± 8.3 years	- pocket depth (PPD) - clinical attachment level (CAL), - gingival index (GI) - gingival bleeding index, - halitosis	SRP with aPDT	SRP alone	- 655 nm with a CW output power of 1 W - The photosensitizer: methylene blue	Chronic periodontitis	- PPD and CAL showed statistically significant reduction in the test group at 3 months and 6 months - gingival index and gingival bleeding index reduction in the test group - reduction of the halitosis in the test group
9	Akiko Ichinose-Tsuno, 2014 [42]	11	28.0 ± 2.3 years	- Level of Streptococcus oralis	The right or left mandibular premolars were randomly assigned to the treatment (with aPDT)	The right or left mandibular premolars were randomly assigned to the treatment (without aPDT)	A combination of 500 or 1000 μg/mL toluidine blue O (TBO) and LED irradiation for 20 s - The photosensitizer: toluidine blue O (TBO)	Health of oral cavity	- significantly decreased the number of Streptococcus oralis
10	Abdulaziz M Albaker [43]	24	aPDT + OFD group 58.4 ± 8.0; OFD group 61.5 ± 9.9	- Peri-implant plaque index (PI) - Bleeding on probing (BOP) - Pocket depth (PD) - Marginal bone level (MBL)	- Patients with peri-implantitis receiving aPDT with OFD (open flap debridement)	- Patients with periimplantitis receiving OFD alone	A diode laser of 670 nanometers at 150 milliwatts with optic fibre diameter 0.06 mm.	Peri-implantitis	At 6 months, aPDT and OFD significantly reduced peri-implant PI, BOP, PD and MBL.

**Table 2:** Risk of bias of randomized clinical trials, assessed through the ROB2 tool [35].

Study	The Randomization Process	Deviations from the Intended Interventions	Missing Outcome Date	Measurement of Outcome Data	Selection of the Reported Result
Seérgio H. L. Martins et al., 2017 [38]	Low risk	Low risk	Low risk	Low risk	Low risk
Tuna Kaplan et al., 2021 [44]	Low risk	High risk	Low risk	Low risk	Low risk
Fotios Katsikanis et al., 2019 [4]	Some concern	Some concern	Low risk	Low risk	Low risk
Subasree Soundarajan 2022 [39]	Low risk	Low risk	Low risk	Low risk	Low risk
Zoran Arsic et al., 2022 [2]	Some concern	Some concern	Low risk	Low risk	Low risk
Greta Hill et al., 2019 [40]	Low risk	Low risk	Low risk	Low risk	Low risk
Betsy Joseph et al., 2014 [41]	Low risk	Some concern	Low risk	Low risk	Low risk
Akiko Ichinose-Tsuno et al., 2014 [42]	Low risk	Low risk	Low risk	Low risk	Some concern
Marco Annunziata et al., 2023 [36]	Low risk	High risk	High risk	High risk	Some concern
Abdulaziz M Albaker 2018 [43]	Low risk	Low risk	Low risk	Low risk	Low risk

## Data Availability

No new data were created in this study. Data sharing is not applicable for this study.
